# Tetra­butyl­ammonium 2-[2,5-dimethyl-3-(4-nitro­phen­yl)-2,3-dihydro-1,2,4-oxadiazo­lium-4-yl]nona­hydro-*closo*-deca­borate

**DOI:** 10.1107/S1600536812044984

**Published:** 2012-11-03

**Authors:** Aleksey L. Mindich, Anna V. Pavlishchuk, Nadezhda A. Bokach, Galina L. Starova, Konstantin Yu. Zhizhin

**Affiliations:** a Department of Chemistry, Saint-Petersburg State University, Universitetsky Pr. 26, 198504 Stary Petergof, Russian Federation; b Department of Chemistry, Kiev National Taras Shevchenko University, Volodymyrska str. 62, Kiev 01601, Ukraine; c N. S. Kurnakov Institute of General and Inorganic Chemistry, Russian Academy of Sciences, 31 Leninsky Pr., 119991 Moscow, Russian Federation

## Abstract

The title ionic compound, C_16_H_36_N^+^·C_10_H_20_B_10_N_3_O_3_^−^, consists of a tetra­butyl­ammonium cation and a *closo*-deca­borate cluster anion, which is bound to the substituted 2,3-dihydro-1,2,4-oxadiazole ring through a B—N bond [1.540 (2) Å]. The distances between connected B atoms in the deca­borate cluster range from 1.689 (2) to 1.844 (2) Å. The 2,3-dihydro-1,2,4-oxadiazole ring adopts an envelope conformation with the N atom as the flap atom.

## Related literature

For related structures and background, see: Mindich *et al.* (2012 ▶). For examples of substituted 1,2,4-oxadiazo­les, their complexes and properties, see: Kritchenkov *et al.* (2012 ▶); Bokach *et al.* (2011*a* ▶,*b* ▶); Makarycheva-Mikhailova *et al.* (2007 ▶); Kukushkin & Pombeiro (2002 ▶); Kritchenkov *et al.* (2011 ▶); Bokach (2010 ▶); Sivaev *et al.* (2002 ▶, 2008 ▶). For propeties and structure examples of boron clusters, see: Dash *et al.* (2011 ▶); Dou *et al.* (1994 ▶).
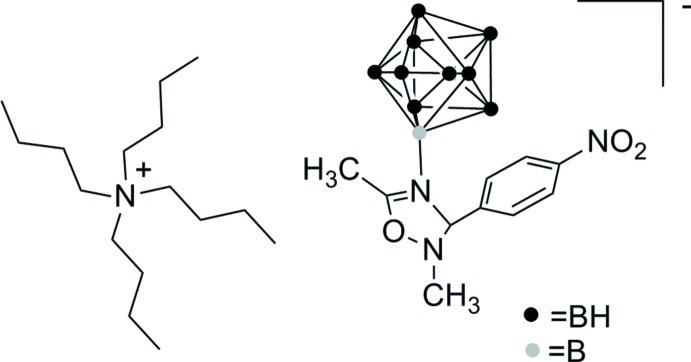


## Experimental

### 

#### Crystal data

C_16_H_36_N^+^·C_10_H_20_B_10_N_3_O_3_^−^
*M* *_r_* = 580.85
Monoclinic, 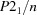

*a* = 8.7356 (4) Å
*b* = 15.8691 (6) Å
*c* = 24.7015 (10) Å
β = 98.206 (1)°
*V* = 3389.2 (2) Å^3^
*Z* = 4
Mo *K*α radiation
μ = 0.07 mm^−1^
*T* = 100 K
0.35 × 0.25 × 0.20 mm

#### Data collection

Bruker Kappa APEXII DUO CCD diffractometer
Absorption correction: multi-scan (*SADABS*; Bruker, 2005 ▶) *T*_min_ = 0.161, *T*_max_ = 0.374
35977 measured reflections
10868 independent reflections
6806 reflections with *I* > 2σ(*I*)
*R*_int_ = 0.086

#### Refinement

*R*[*F* ^2^ > 2σ(*F* ^2^)] = 0.054
*wR*(*F* ^2^) = 0.142
*S* = 0.94
10868 reflections
388 parameters
H-atom parameters constrained
Δρ_max_ = 0.55 e Å^−3^
Δρ_min_ = −0.51 e Å^−3^

### 

Data collection: *APEX2* (Bruker, 2005 ▶); cell refinement: *SAINT* (Bruker, 2005 ▶); data reduction: *SAINT*; program(s) used to solve structure: *SIR92* (Altomare *et al.*, 1993 ▶); program(s) used to refine structure: *SHELXL97* (Sheldrick, 2008 ▶); molecular graphics: *SHELXTL* (Sheldrick, 2008 ▶); software used to prepare material for publication: *SHELXTL*.

## Supplementary Material

Crystal structure: contains datablock(s) I, global. DOI: 10.1107/S1600536812044984/aa2067sup1.cif

Structure factors: contains datablock(s) I. DOI: 10.1107/S1600536812044984/aa2067Isup2.hkl

Additional supplementary materials:  crystallographic information; 3D view; checkCIF report

## Figures and Tables

**Fig. 1.**
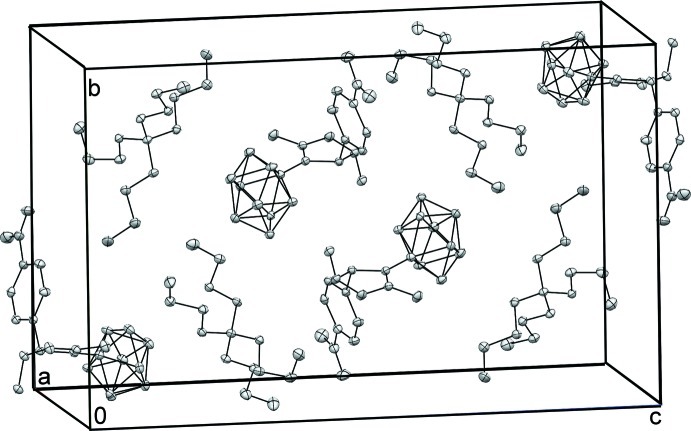
A packing view of the title compound.

**Fig. 2.**
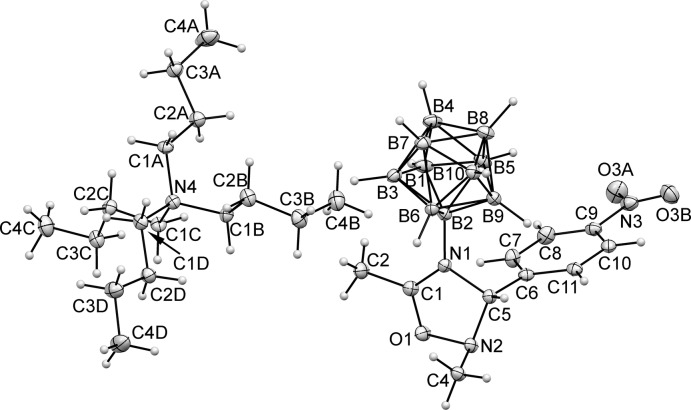
A view of the title compound with the atomic numbering scheme (thermal ellipsoids are drawn at the 50% probability level.

## supplementary crystallographic information

### Comment

In the past decade, a great attention has been paid to electrophilic activation of the C≡N bond in nitriles (Bokach, 2010; Bokach *et al.*, 2011*a*; Bokach *et al.*, 2011*b*; Kritchenkov *et al.*, 2011; Kritchenkov *et al.*, 2012; Kukushkin & Pombeiro, 2002; Makarycheva-Mikhailova *et al.*, 2007). In a addition an interest in compounds which contain borane clusters is caused by their potential application in medicine, homogeneous catalysis, for non-linear optics and luminescent materials creation, as potential metal extractants, agents for anion recognition and building blocks for coordination polymers creation, *etc* (Dash *et al.*, 2011). Despite boron clusters are known near the century, examples of coordination compounds based thereon are sparse. Therefore the synthesis of new ligand systems based on borane clusters and investigation of their ability of complex formation are of particular interest. In the framework of our project focused on reactivity of the activated C≡N group in nitrilium derivatives of *closo*-decaborate clusters (Mindich *et al.*, 2012), the substituted borylated 2,3-dihydro-1,2,4-oxadiazole (Scheme 1) was characterized by a single-crystal X-ray diffraction.

Each unit cell (Fig. 1) of the title compound, [*n*-Bu_4_N]^+^[C_10_H_20_B_10_O_3_N_3_]^-^, consists of four anions and four cations. So far as there are no hydrogen bonds in the title compound, it should be mentioned that crystal packing is generally caused by electrostatic interactions between cations and anions. In the cation of the title compound, the alkyl chains of [*n*-Bu_4_N]^+^ are always in *anti* or *gauche* conformations and all bond lengths are close to those reported in the literature (Sivaev *et al.*, 2008).

The anionic part consists of a substituted 2,3-dihydro-1,2,4-oxadiazole ring bound to the *closo*-decaborate cluster *via* the N1 atom [*d*(B2—N1) = 1.540 (3) Å] (Fig. 2). This bond is longer as compared to the starting nitrilium *closo*-decaborate (1.515 (5) Å) (Dou *et al.*, 1994) and is equal, within 3*σ*, to those in the previously described *closo*-decaborate clusters functionalized with substituted 2,3-dihydro-1,2,4-oxadiazoles (1.5438 (18)–1.546 (3) Å) (Mindich *et al.*, 2012).

Ten boron atoms in the decaborate cluster are connected between each other in such way, that they form a bicapped square antiprism. Each of two boron atoms (B1 and B10), which are situated in the vertex of observed polyhedron, are surrounded by four boron atoms. Bond distances between B1 or B10 and connected with them boron atoms (B2 – B5 for B1 and B6 – B9 for B10) range from 1.689 (2) Å to 1.708 (2) Å. Each of the boron atoms B2 – B9 are surrounded by five boron atoms. Bond distances between connected boron atoms B2 – B9 vary from 1.806 (2) Å to 1.844 (2) Å, what is on 0.117 – 0.136 Å longer than for B1—B(2–5) and B10—B(6–9) bonds. In the cluster, the B—B bond distances and angles are typical for 2-substituted-nonahydro-*closo*-decaborates (Dou *et al.*, 1994; Sivaev *et al.*, 2002).

The double bond in 1,2,4-oxadiazole in the title compound is observed between atoms N1 and C1 [*d* = 1.2886 (16) Å]. The 2,3-dihydro-1,2,4-oxadiazole ring adopts an envelope conformation with N2 being the flap atom. Deviation of N2 from the plane O1C1N1C5 is equal to 0.327 (2) Å. 1,2,4-oxadiazole contains two methyl groups in the 2-nd and 5-th positions and *p*-nitrophenyl in the 3-rd position. The angle between mean planes of substituted 1,2,4-oxadiazole ring O1C1N1C5 and phenyl ring C6C7C8C9C10C11 is equal to 86.35 (18)°. The geometrical parameters of the 2,3-dihydro-1,2,4-oxadiazole rings are the same, within 3*σ*, as those in the previously described borylated 2,3-dihydro-1,2,4-oxadiazoles (Mindich *et al.*, 2012). All bonds and angles are of normal values.

### Experimental

Title compound was synthesized and isolated as pure solid by the described method (Mindich *et al.*, 2012). The crystal was obtained by a slow evaporation of a methanol solution of the title compound. XRD study was carried out in the X-ray Diffraction Centre of St.Petersburg State University.

### Refinement

Refinement of F^2^ against all reflections. The weighted *R*-factor *wR* and goodness of fit S are based on F^2^, conventional *R*-factors *R* are based on F, with F set to zero for negative F^2^. The threshold expression of F^2^ > 2*σ*(F^2^) is used only for calculating *R*-factors(gt) *etc*. and is not relevant to the choice of reflections for refinement. *R*-factors based on F^2^ are statistically about twice as large as those based on F, and R– factors based on all data will be even larger.

### Crystal data

**Table:**

C_16_H_36_N^+^·C_10_H_20_B_10_N_3_O_3_^−^	*F*(000) = 1256
*M**_r_* = 580.85	*D*_x_ = 1.138 Mg m^−^^3^
Monoclinic, *P*2_1_/*n*	Mo *K*α radiation, λ = 0.71073 Å
Hall symbol: -P 2yn	Cell parameters from 6524 reflections
*a* = 8.7356 (4) Å	θ = 2.6–30.8°
*b* = 15.8691 (6) Å	µ = 0.07 mm^−^^1^
*c* = 24.7015 (10) Å	*T* = 100 K
β = 98.206 (1)°	Block, colorless
*V* = 3389.2 (2) Å^3^	0.35 × 0.25 × 0.20 mm
*Z* = 4	

### Data collection

**Table:**

Bruker Kappa APEXII DUO CCD diffractometer	10868 independent reflections
Radiation source: fine-focus sealed tube	6806 reflections with *I* > 2σ(*I*)
Graphite monochromator	*R*_int_ = 0.086
φ and ω scans	θ_max_ = 31.2°, θ_min_ = 2.1°
Absorption correction: multi-scan (*SADABS*; Bruker, 2005)	*h* = −12→6
*T*_min_ = 0.161, *T*_max_ = 0.374	*k* = −23→20
35977 measured reflections	*l* = −34→35

### Refinement

**Table:**

Refinement on *F*^2^	Primary atom site location: structure-invariant direct methods
Least-squares matrix: full	Secondary atom site location: difference Fourier map
*R*[*F*^2^ > 2σ(*F*^2^)] = 0.054	Hydrogen site location: inferred from neighbouring sites
*wR*(*F*^2^) = 0.142	H-atom parameters constrained
*S* = 0.94	*w* = 1/[σ^2^(*F*_o_^2^) + (0.0609*P*)^2^] where *P* = (*F*_o_^2^ + 2*F*_c_^2^)/3
10868 reflections	(Δ/σ)_max_ < 0.001
388 parameters	Δρ_max_ = 0.55 e Å^−^^3^
0 restraints	Δρ_min_ = −0.51 e Å^−^^3^

### Special details

**Table:**

Geometry. All s.u.'s (except the s.u. in the dihedral angle between two l.s. planes) are estimated using the full covariance matrix. The cell s.u.'s are taken into account individually in the estimation of s.u.'s in distances, angles and torsion angles; correlations between s.u.'s in cell parameters are only used when they are defined by crystal symmetry. An approximate (isotropic) treatment of cell s.u.'s is used for estimating s.u.'s involving l.s. planes.
Refinement. Refinement of *F*^2^ against ALL reflections. The weighted *R*-factor *wR* and goodness of fit *S* are based on *F*^2^, conventional *R*-factors *R* are based on *F*, with *F* set to zero for negative *F*^2^. The threshold expression of *F*^2^ > σ(*F*^2^) is used only for calculating *R*-factors(gt) *etc*. and is not relevant to the choice of reflections for refinement. *R*-factors based on *F*^2^ are statistically about twice as large as those based on *F*, and *R*-factors based on ALL data will be even larger.

### Fractional atomic coordinates and isotropic or equivalent isotropic displacement parameters (Å^2^)

**Table:**

	*x*	*y*	*z*	*U*_iso_*/*U*_eq_	
N4	1.06470 (12)	0.31892 (7)	−0.19722 (4)	0.0152 (2)	
C1A	0.99038 (15)	0.30280 (8)	−0.25556 (5)	0.0166 (3)	
H1AA	1.0716	0.2948	−0.2781	0.020*	
H1AB	0.9323	0.3525	−0.2689	0.020*	
C2A	0.88276 (16)	0.22708 (8)	−0.26301 (6)	0.0189 (3)	
H2AA	0.7830	0.2420	−0.2528	0.023*	
H2AB	0.9257	0.1814	−0.2395	0.023*	
C3A	0.86245 (16)	0.19856 (9)	−0.32259 (6)	0.0211 (3)	
H3AA	0.8326	0.2465	−0.3461	0.025*	
H3AB	0.9605	0.1776	−0.3312	0.025*	
C4A	0.7411 (2)	0.13008 (9)	−0.33407 (6)	0.0311 (4)	
H4AA	0.7311	0.1143	−0.3719	0.047*	
H4AB	0.6437	0.1507	−0.3259	0.047*	
H4AC	0.7718	0.0819	−0.3117	0.047*	
C1B	0.94265 (15)	0.32580 (8)	−0.15940 (5)	0.0164 (3)	
H1BA	0.9944	0.3320	−0.1222	0.020*	
H1BB	0.8847	0.2735	−0.1612	0.020*	
C2B	0.82901 (16)	0.39833 (8)	−0.17181 (6)	0.0198 (3)	
H2BA	0.7641	0.3882	−0.2064	0.024*	
H2BB	0.8854	0.4504	−0.1749	0.024*	
C3B	0.72812 (17)	0.40682 (9)	−0.12650 (6)	0.0232 (3)	
H3BA	0.7946	0.4144	−0.0919	0.028*	
H3BB	0.6649	0.4570	−0.1332	0.028*	
C4B	0.62259 (17)	0.33156 (10)	−0.12169 (7)	0.0273 (3)	
H4BA	0.5635	0.3410	−0.0923	0.041*	
H4BB	0.6841	0.2816	−0.1144	0.041*	
H4BC	0.5537	0.3247	−0.1553	0.041*	
C1C	1.16972 (15)	0.24577 (8)	−0.17577 (5)	0.0168 (3)	
H1CA	1.2302	0.2629	−0.1415	0.020*	
H1CB	1.1051	0.1989	−0.1679	0.020*	
C2C	1.28027 (16)	0.21468 (8)	−0.21379 (6)	0.0182 (3)	
H2CA	1.3361	0.2620	−0.2263	0.022*	
H2CB	1.2223	0.1877	−0.2455	0.022*	
C3C	1.39420 (15)	0.15209 (8)	−0.18342 (6)	0.0194 (3)	
H3CA	1.4461	0.1782	−0.1503	0.023*	
H3CB	1.3380	0.1034	−0.1729	0.023*	
C4C	1.51453 (17)	0.12333 (10)	−0.21844 (7)	0.0282 (3)	
H4CA	1.5831	0.0835	−0.1983	0.042*	
H4CB	1.5727	0.1711	−0.2279	0.042*	
H4CC	1.4636	0.0973	−0.2512	0.042*	
C1D	1.15761 (16)	0.40003 (8)	−0.19808 (6)	0.0172 (3)	
H1DA	1.2440	0.3894	−0.2178	0.021*	
H1DB	1.0925	0.4420	−0.2185	0.021*	
C2D	1.22037 (16)	0.43691 (8)	−0.14269 (6)	0.0194 (3)	
H2DA	1.2882	0.3964	−0.1220	0.023*	
H2DB	1.1355	0.4487	−0.1225	0.023*	
C3D	1.30961 (19)	0.51795 (9)	−0.14965 (6)	0.0260 (3)	
H3DA	1.3931	0.5059	−0.1705	0.031*	
H3DB	1.2410	0.5583	−0.1702	0.031*	
C4D	1.3761 (2)	0.55653 (10)	−0.09510 (6)	0.0320 (4)	
H4DA	1.4314	0.6071	−0.1013	0.048*	
H4DB	1.4455	0.5171	−0.0748	0.048*	
H4DC	1.2936	0.5697	−0.0747	0.048*	
O1	0.54528 (11)	0.16501 (6)	−0.01773 (4)	0.0202 (2)	
N1	0.74065 (12)	0.12499 (6)	0.04378 (4)	0.0144 (2)	
N2	0.66334 (12)	0.13225 (7)	−0.05053 (5)	0.0165 (2)	
C1	0.59955 (15)	0.15096 (8)	0.03466 (6)	0.0168 (3)	
C2	0.49407 (16)	0.17008 (9)	0.07497 (6)	0.0222 (3)	
H2A	0.3957	0.1879	0.0561	0.033*	
H2B	0.5377	0.2143	0.0989	0.033*	
H2C	0.4805	0.1205	0.0961	0.033*	
C4	0.60748 (16)	0.04754 (8)	−0.06730 (6)	0.0182 (3)	
H4A	0.5161	0.0521	−0.0938	0.027*	
H4B	0.5837	0.0171	−0.0359	0.027*	
H4C	0.6862	0.0180	−0.0831	0.027*	
C5	0.80327 (15)	0.12692 (8)	−0.00925 (5)	0.0148 (3)	
H5	0.8579	0.0741	−0.0140	0.018*	
C6	0.91407 (15)	0.20041 (8)	−0.00974 (5)	0.0156 (3)	
C7	0.87874 (16)	0.28045 (8)	0.00783 (6)	0.0206 (3)	
H7	0.7820	0.2908	0.0180	0.025*	
C8	0.98651 (17)	0.34482 (9)	0.01034 (6)	0.0222 (3)	
H8	0.9630	0.3986	0.0216	0.027*	
C9	1.13015 (16)	0.32699 (8)	−0.00431 (5)	0.0181 (3)	
C10	1.16758 (15)	0.24852 (8)	−0.02307 (5)	0.0172 (3)	
H10	1.2641	0.2386	−0.0336	0.021*	
C11	1.05790 (15)	0.18518 (8)	−0.02585 (5)	0.0163 (3)	
H11	1.0804	0.1321	−0.0386	0.020*	
N3	1.24830 (15)	0.39366 (8)	0.00029 (5)	0.0226 (3)	
O3A	1.21039 (14)	0.46527 (6)	0.01226 (5)	0.0338 (3)	
O3B	1.37898 (12)	0.37441 (7)	−0.00819 (5)	0.0314 (3)	
B1	0.84962 (19)	0.16988 (10)	0.15185 (7)	0.0202 (3)	
H12	0.7988	0.2324	0.1557	0.030*	
B2	0.84165 (17)	0.10294 (9)	0.09822 (6)	0.0152 (3)	
B3	0.78915 (18)	0.07008 (10)	0.16335 (6)	0.0185 (3)	
H13	0.6764	0.0609	0.1777	0.028*	
B4	0.97278 (19)	0.11400 (10)	0.19715 (7)	0.0219 (3)	
H14	1.0038	0.1399	0.2375	0.033*	
B5	1.02284 (18)	0.14771 (10)	0.13054 (7)	0.0190 (3)	
H15	1.0997	0.2006	0.1211	0.028*	
B6	0.85624 (17)	−0.00739 (9)	0.11691 (6)	0.0151 (3)	
H16	0.7707	−0.0506	0.0929	0.023*	
B7	0.94918 (18)	0.00066 (10)	0.18820 (6)	0.0185 (3)	
H17	0.9411	−0.0380	0.2246	0.028*	
B8	1.11443 (18)	0.05523 (10)	0.16491 (7)	0.0192 (3)	
H18	1.2426	0.0601	0.1834	0.029*	
B9	1.02020 (17)	0.04734 (9)	0.09450 (6)	0.0153 (3)	
H19	1.0644	0.0470	0.0534	0.023*	
B10	1.04235 (17)	−0.03695 (9)	0.13722 (6)	0.0163 (3)	
H20	1.0971	−0.0992	0.1344	0.024*	

### Atomic displacement parameters (Å^2^)

**Table:**

	*U*^11^	*U*^22^	*U*^33^	*U*^12^	*U*^13^	*U*^23^
N4	0.0155 (5)	0.0132 (5)	0.0167 (6)	0.0006 (4)	0.0018 (5)	0.0020 (4)
C1A	0.0177 (6)	0.0160 (6)	0.0154 (6)	0.0003 (5)	−0.0001 (5)	0.0019 (5)
C2A	0.0218 (7)	0.0156 (6)	0.0190 (7)	−0.0022 (6)	0.0023 (6)	0.0006 (5)
C3A	0.0226 (7)	0.0204 (7)	0.0204 (7)	−0.0004 (6)	0.0031 (6)	−0.0005 (6)
C4A	0.0443 (10)	0.0247 (8)	0.0240 (8)	−0.0107 (7)	0.0033 (8)	−0.0059 (6)
C1B	0.0174 (6)	0.0148 (6)	0.0172 (6)	0.0002 (5)	0.0036 (5)	0.0017 (5)
C2B	0.0182 (6)	0.0153 (6)	0.0259 (8)	0.0010 (5)	0.0036 (6)	0.0030 (6)
C3B	0.0204 (7)	0.0210 (7)	0.0285 (8)	0.0039 (6)	0.0043 (6)	−0.0015 (6)
C4B	0.0222 (7)	0.0287 (8)	0.0319 (9)	0.0020 (6)	0.0066 (7)	0.0055 (7)
C1C	0.0170 (6)	0.0151 (6)	0.0179 (7)	0.0027 (5)	0.0012 (5)	0.0046 (5)
C2C	0.0197 (6)	0.0171 (6)	0.0182 (7)	0.0007 (6)	0.0037 (6)	0.0026 (5)
C3C	0.0166 (6)	0.0169 (6)	0.0246 (7)	0.0005 (5)	0.0033 (6)	0.0035 (5)
C4C	0.0217 (7)	0.0273 (8)	0.0367 (9)	0.0038 (6)	0.0073 (7)	0.0018 (7)
C1D	0.0183 (6)	0.0134 (6)	0.0196 (7)	−0.0020 (5)	0.0019 (6)	0.0032 (5)
C2D	0.0203 (7)	0.0185 (7)	0.0194 (7)	−0.0023 (6)	0.0027 (6)	−0.0001 (5)
C3D	0.0312 (8)	0.0235 (7)	0.0225 (8)	−0.0100 (7)	0.0008 (7)	0.0012 (6)
C4D	0.0419 (10)	0.0286 (8)	0.0258 (8)	−0.0187 (8)	0.0067 (8)	−0.0048 (7)
O1	0.0172 (5)	0.0238 (5)	0.0190 (5)	0.0083 (4)	0.0009 (4)	−0.0019 (4)
N1	0.0149 (5)	0.0131 (5)	0.0152 (5)	0.0010 (4)	0.0022 (4)	−0.0014 (4)
N2	0.0140 (5)	0.0173 (6)	0.0181 (6)	0.0036 (5)	0.0019 (5)	−0.0028 (4)
C1	0.0178 (6)	0.0140 (6)	0.0182 (7)	0.0022 (5)	0.0014 (5)	−0.0010 (5)
C2	0.0179 (6)	0.0272 (8)	0.0218 (7)	0.0063 (6)	0.0035 (6)	−0.0033 (6)
C4	0.0161 (6)	0.0186 (7)	0.0190 (7)	−0.0002 (5)	0.0001 (5)	−0.0032 (5)
C5	0.0148 (6)	0.0139 (6)	0.0155 (6)	0.0022 (5)	0.0015 (5)	−0.0004 (5)
C6	0.0170 (6)	0.0145 (6)	0.0144 (6)	0.0018 (5)	−0.0002 (5)	0.0003 (5)
C7	0.0199 (7)	0.0166 (6)	0.0261 (8)	0.0025 (6)	0.0060 (6)	−0.0010 (6)
C8	0.0274 (7)	0.0151 (6)	0.0246 (8)	0.0013 (6)	0.0059 (6)	−0.0019 (6)
C9	0.0213 (7)	0.0166 (6)	0.0155 (6)	−0.0045 (6)	−0.0005 (6)	0.0000 (5)
C10	0.0163 (6)	0.0201 (7)	0.0146 (6)	0.0009 (5)	0.0006 (5)	0.0021 (5)
C11	0.0176 (6)	0.0153 (6)	0.0154 (6)	0.0030 (5)	0.0004 (5)	0.0001 (5)
N3	0.0275 (6)	0.0213 (6)	0.0184 (6)	−0.0065 (5)	0.0014 (5)	0.0000 (5)
O3A	0.0450 (7)	0.0185 (5)	0.0398 (7)	−0.0090 (5)	0.0124 (6)	−0.0067 (5)
O3B	0.0245 (6)	0.0327 (6)	0.0367 (7)	−0.0082 (5)	0.0033 (5)	−0.0027 (5)
B1	0.0221 (8)	0.0176 (7)	0.0194 (8)	0.0033 (6)	−0.0020 (7)	−0.0053 (6)
B2	0.0143 (7)	0.0152 (7)	0.0159 (7)	0.0005 (6)	0.0012 (6)	−0.0001 (6)
B3	0.0192 (7)	0.0227 (8)	0.0138 (7)	0.0045 (6)	0.0025 (6)	−0.0015 (6)
B4	0.0229 (8)	0.0223 (8)	0.0190 (8)	0.0051 (7)	−0.0020 (7)	−0.0069 (6)
B5	0.0171 (7)	0.0145 (7)	0.0236 (8)	0.0001 (6)	−0.0028 (6)	−0.0043 (6)
B6	0.0151 (7)	0.0151 (7)	0.0151 (7)	−0.0008 (6)	0.0020 (6)	−0.0015 (6)
B7	0.0190 (7)	0.0197 (8)	0.0163 (7)	0.0034 (6)	0.0007 (6)	0.0012 (6)
B8	0.0165 (7)	0.0188 (7)	0.0206 (8)	0.0019 (6)	−0.0030 (6)	−0.0031 (6)
B9	0.0142 (7)	0.0136 (7)	0.0179 (7)	0.0003 (6)	0.0015 (6)	−0.0015 (6)
B10	0.0158 (7)	0.0154 (7)	0.0176 (7)	0.0020 (6)	0.0015 (6)	−0.0001 (6)

### Geometric parameters (Å, º)

**Table:**

N4—C1A	1.5154 (17)	B2—B6	1.810 (2)
N4—C1B	1.5184 (15)	B2—B3	1.811 (2)
N4—C1D	1.5234 (16)	B2—B5	1.813 (2)
N4—C1C	1.5268 (16)	B3—B7	1.816 (2)
C1A—C2A	1.5206 (18)	B3—B6	1.834 (2)
C2A—C3A	1.5255 (19)	B3—B4	1.837 (2)
C3A—C4A	1.516 (2)	B4—B7	1.820 (2)
C1B—C2B	1.5222 (18)	B4—B8	1.821 (2)
C2B—C3B	1.5261 (18)	B4—B5	1.841 (2)
C3B—C4B	1.524 (2)	B5—B8	1.822 (2)
C1C—C2C	1.5221 (17)	B5—B9	1.823 (2)
C2C—C3C	1.5254 (19)	B6—B10	1.698 (2)
C3C—C4C	1.5235 (18)	B6—B9	1.827 (2)
C1D—C2D	1.5168 (19)	B6—B7	1.835 (2)
C2D—C3D	1.5263 (18)	B7—B10	1.702 (2)
C3D—C4D	1.518 (2)	B7—B8	1.844 (2)
O1—C1	1.3319 (16)	B8—B10	1.697 (2)
O1—N2	1.4929 (13)	B8—B9	1.819 (2)
N1—C1	1.2886 (16)	B9—B10	1.698 (2)
N1—C5	1.4903 (15)	C2—H2A	0.9600
N1—B2	1.5397 (19)	C2—H2B	0.9600
N2—C4	1.4694 (16)	C2—H2C	0.9600
N2—C5	1.4781 (17)	C4—H4A	0.9600
C1—C2	1.4815 (17)	C4—H4B	0.9600
C5—C6	1.5167 (18)	C4—H4C	0.9600
C6—C7	1.3910 (18)	C5—H5	0.9800
C6—C11	1.3923 (17)	C7—H7	0.9300
C7—C8	1.3846 (19)	C8—H8	0.9300
C8—C9	1.3839 (18)	C10—H10	0.9300
C9—C10	1.3841 (18)	C11—H11	0.9300
C9—N3	1.4710 (17)	B1—H12	1.0971
C10—C11	1.3836 (18)	B3—H13	1.1035
N3—O3B	1.2283 (15)	B4—H14	1.0770
N3—O3A	1.2315 (15)	B5—H15	1.1202
B1—B4	1.689 (2)	B6—H16	1.1197
B1—B2	1.692 (2)	B7—H17	1.0992
B1—B3	1.706 (2)	B8—H18	1.1497
B1—B5	1.708 (2)	B9—H19	1.1365
B2—B9	1.806 (2)	B10—H20	1.1034
			
C1A—N4—C1B	110.74 (10)	B8—B4—B3	102.17 (10)
C1A—N4—C1D	106.63 (9)	B1—B4—B5	57.68 (9)
C1B—N4—C1D	111.72 (9)	B7—B4—B5	102.46 (10)
C1A—N4—C1C	110.87 (10)	B8—B4—B5	59.68 (8)
C1B—N4—C1C	106.32 (9)	B3—B4—B5	90.43 (10)
C1D—N4—C1C	110.63 (10)	B1—B5—B2	57.33 (9)
N4—C1A—C2A	114.97 (10)	B1—B5—B8	111.81 (11)
C1A—C2A—C3A	109.64 (10)	B2—B5—B8	100.79 (10)
C4A—C3A—C2A	112.03 (11)	B1—B5—B9	112.36 (11)
N4—C1B—C2B	115.18 (10)	B2—B5—B9	59.55 (8)
C1B—C2B—C3B	110.33 (11)	B8—B5—B9	59.87 (8)
C4B—C3B—C2B	114.02 (12)	B1—B5—B4	56.68 (9)
C2C—C1C—N4	115.79 (10)	B2—B5—B4	88.61 (10)
C1C—C2C—C3C	109.55 (11)	B8—B5—B4	59.61 (9)
C4C—C3C—C2C	111.75 (11)	B9—B5—B4	101.21 (10)
C2D—C1D—N4	115.98 (10)	B10—B6—B2	111.85 (11)
C1D—C2D—C3D	110.38 (11)	B10—B6—B9	57.44 (8)
C4D—C3D—C2D	112.14 (12)	B2—B6—B9	59.52 (8)
C1—O1—N2	106.93 (9)	B10—B6—B3	112.24 (11)
C1—N1—C5	107.55 (11)	B2—B6—B3	59.61 (8)
C1—N1—B2	129.79 (11)	B9—B6—B3	101.84 (10)
C5—N1—B2	122.41 (10)	B10—B6—B7	57.44 (9)
C4—N2—C5	110.54 (10)	B2—B6—B7	100.53 (10)
C4—N2—O1	104.14 (9)	B9—B6—B7	89.95 (10)
C5—N2—O1	102.30 (9)	B3—B6—B7	59.35 (8)
N1—C1—O1	114.99 (11)	B10—B7—B3	112.88 (11)
N1—C1—C2	128.25 (13)	B10—B7—B4	112.24 (11)
O1—C1—C2	116.72 (12)	B3—B7—B4	60.67 (9)
N2—C5—N1	103.67 (9)	B10—B7—B6	57.21 (8)
N2—C5—C6	114.61 (10)	B3—B7—B6	60.28 (8)
N1—C5—C6	109.72 (10)	B4—B7—B6	102.25 (10)
C7—C6—C11	119.81 (12)	B10—B7—B8	57.02 (8)
C7—C6—C5	121.97 (11)	B3—B7—B8	102.05 (10)
C11—C6—C5	118.14 (11)	B4—B7—B8	59.59 (8)
C8—C7—C6	120.53 (12)	B6—B7—B8	89.55 (9)
C9—C8—C7	118.26 (12)	B10—B8—B9	57.60 (9)
C8—C9—C10	122.54 (13)	B10—B8—B4	112.42 (11)
C8—C9—N3	119.02 (12)	B9—B8—B4	102.14 (11)
C10—C9—N3	118.45 (12)	B10—B8—B5	113.16 (12)
C11—C10—C9	118.38 (12)	B9—B8—B5	60.09 (8)
C10—C11—C6	120.43 (12)	B4—B8—B5	60.71 (9)
O3B—N3—O3A	123.98 (12)	B10—B8—B7	57.27 (8)
O3B—N3—C9	117.83 (12)	B9—B8—B7	89.91 (10)
O3A—N3—C9	118.20 (12)	B4—B8—B7	59.54 (9)
B4—B1—B2	98.06 (11)	B5—B8—B7	102.26 (10)
B4—B1—B3	65.50 (10)	B10—B9—B2	112.08 (10)
B2—B1—B3	64.43 (9)	B10—B9—B8	57.59 (9)
B4—B1—B5	65.64 (10)	B2—B9—B8	101.18 (10)
B2—B1—B5	64.45 (9)	B10—B9—B5	113.11 (11)
B3—B1—B5	99.74 (11)	B2—B9—B5	59.94 (8)
N1—B2—B1	119.71 (11)	B8—B9—B5	60.04 (9)
N1—B2—B9	117.26 (10)	B10—B9—B6	57.44 (8)
B1—B2—B9	114.04 (11)	B2—B9—B6	59.78 (8)
N1—B2—B6	116.95 (11)	B8—B9—B6	90.59 (9)
B1—B2—B6	114.46 (11)	B5—B9—B6	102.89 (10)
B9—B2—B6	60.70 (8)	B8—B10—B9	64.80 (9)
N1—B2—B3	130.92 (11)	B8—B10—B6	99.52 (11)
B1—B2—B3	58.18 (9)	B9—B10—B6	65.12 (9)
B9—B2—B3	103.57 (10)	B8—B10—B7	65.71 (9)
B6—B2—B3	60.84 (8)	B9—B10—B7	99.18 (11)
N1—B2—B5	130.61 (11)	B6—B10—B7	65.35 (9)
B1—B2—B5	58.22 (9)	C1—C2—H2A	109.5
B9—B2—B5	60.51 (8)	C1—C2—H2B	109.5
B6—B2—B5	103.97 (10)	H2A—C2—H2B	109.5
B3—B2—B5	92.16 (10)	C1—C2—H2C	109.5
B1—B3—B2	57.39 (9)	H2A—C2—H2C	109.5
B1—B3—B7	112.18 (11)	H2B—C2—H2C	109.5
B2—B3—B7	101.22 (10)	N2—C4—H4A	109.5
B1—B3—B6	112.57 (10)	N2—C4—H4B	109.5
B2—B3—B6	59.56 (8)	H4A—C4—H4B	109.5
B7—B3—B6	60.37 (8)	N2—C4—H4C	109.5
B1—B3—B4	56.80 (9)	H4A—C4—H4C	109.5
B2—B3—B4	88.80 (10)	H4B—C4—H4C	109.5
B7—B3—B4	59.76 (9)	N2—C5—H5	109.5
B6—B3—B4	101.67 (10)	N1—C5—H5	109.5
B1—B4—B7	112.83 (12)	C6—C5—H5	109.5
B1—B4—B8	112.80 (12)	C8—C7—H7	119.7
B7—B4—B8	60.86 (8)	C6—C7—H7	119.7
B1—B4—B3	57.70 (9)	C9—C8—H8	120.9
B7—B4—B3	59.57 (9)	C7—C8—H8	120.9
			
C1B—N4—C1A—C2A	−54.33 (14)	B7—B3—B6—B2	−126.43 (11)
C1D—N4—C1A—C2A	−176.07 (11)	B4—B3—B6—B2	−81.59 (10)
C1C—N4—C1A—C2A	63.44 (13)	B1—B3—B6—B9	20.73 (15)
N4—C1A—C2A—C3A	−159.65 (11)	B2—B3—B6—B9	43.63 (9)
C1A—C2A—C3A—C4A	−172.99 (12)	B7—B3—B6—B9	−82.80 (10)
C1A—N4—C1B—C2B	−62.33 (14)	B4—B3—B6—B9	−37.96 (12)
C1D—N4—C1B—C2B	56.37 (15)	B1—B3—B6—B7	103.53 (13)
C1C—N4—C1B—C2B	177.15 (11)	B2—B3—B6—B7	126.43 (11)
N4—C1B—C2B—C3B	−171.56 (11)	B4—B3—B6—B7	44.84 (10)
C1B—C2B—C3B—C4B	−64.75 (16)	B1—B3—B7—B10	−80.78 (14)
C1A—N4—C1C—C2C	47.08 (14)	B2—B3—B7—B10	−21.61 (14)
C1B—N4—C1C—C2C	167.50 (11)	B6—B3—B7—B10	23.40 (11)
C1D—N4—C1C—C2C	−71.01 (14)	B4—B3—B7—B10	−103.54 (12)
N4—C1C—C2C—C3C	170.86 (11)	B1—B3—B7—B4	22.76 (11)
C1C—C2C—C3C—C4C	−176.20 (12)	B2—B3—B7—B4	81.93 (10)
C1A—N4—C1D—C2D	169.95 (11)	B6—B3—B7—B4	126.94 (10)
C1B—N4—C1D—C2D	48.84 (15)	B1—B3—B7—B6	−104.18 (12)
C1C—N4—C1D—C2D	−69.41 (13)	B2—B3—B7—B6	−45.01 (9)
N4—C1D—C2D—C3D	−179.60 (11)	B4—B3—B7—B6	−126.94 (10)
C1D—C2D—C3D—C4D	−179.35 (13)	B1—B3—B7—B8	−21.72 (14)
C1—O1—N2—C4	96.65 (11)	B2—B3—B7—B8	37.46 (12)
C1—O1—N2—C5	−18.51 (12)	B6—B3—B7—B8	82.46 (10)
C5—N1—C1—O1	5.28 (15)	B4—B3—B7—B8	−44.48 (10)
B2—N1—C1—O1	179.62 (11)	B1—B4—B7—B10	81.48 (14)
C5—N1—C1—C2	−172.03 (13)	B8—B4—B7—B10	−22.80 (11)
B2—N1—C1—C2	2.3 (2)	B3—B4—B7—B10	104.60 (12)
N2—O1—C1—N1	8.72 (15)	B5—B4—B7—B10	21.55 (15)
N2—O1—C1—C2	−173.65 (11)	B1—B4—B7—B3	−23.12 (11)
C4—N2—C5—N1	−89.84 (11)	B8—B4—B7—B3	−127.40 (11)
O1—N2—C5—N1	20.57 (11)	B5—B4—B7—B3	−83.05 (11)
C4—N2—C5—C6	150.60 (10)	B1—B4—B7—B6	22.14 (14)
O1—N2—C5—C6	−98.99 (11)	B8—B4—B7—B6	−82.14 (10)
C1—N1—C5—N2	−16.91 (13)	B3—B4—B7—B6	45.26 (9)
B2—N1—C5—N2	168.24 (10)	B5—B4—B7—B6	−37.79 (12)
C1—N1—C5—C6	105.94 (12)	B1—B4—B7—B8	104.28 (13)
B2—N1—C5—C6	−68.91 (14)	B3—B4—B7—B8	127.40 (11)
N2—C5—C6—C7	70.62 (16)	B5—B4—B7—B8	44.35 (10)
N1—C5—C6—C7	−45.51 (17)	B2—B6—B7—B10	−109.30 (11)
N2—C5—C6—C11	−112.55 (13)	B9—B6—B7—B10	−50.36 (9)
N1—C5—C6—C11	131.33 (12)	B3—B6—B7—B10	−154.20 (11)
C11—C6—C7—C8	−1.1 (2)	B10—B6—B7—B3	154.20 (11)
C5—C6—C7—C8	175.64 (13)	B2—B6—B7—B3	44.90 (9)
C6—C7—C8—C9	−0.9 (2)	B9—B6—B7—B3	103.84 (10)
C7—C8—C9—C10	2.3 (2)	B10—B6—B7—B4	108.71 (11)
C7—C8—C9—N3	−177.48 (13)	B2—B6—B7—B4	−0.58 (11)
C8—C9—C10—C11	−1.7 (2)	B9—B6—B7—B4	58.35 (10)
N3—C9—C10—C11	178.13 (12)	B3—B6—B7—B4	−45.48 (9)
C9—C10—C11—C6	−0.43 (19)	B10—B6—B7—B8	50.02 (9)
C7—C6—C11—C10	1.8 (2)	B2—B6—B7—B8	−59.27 (10)
C5—C6—C11—C10	−175.09 (12)	B9—B6—B7—B8	−0.34 (9)
C8—C9—N3—O3B	173.56 (13)	B3—B6—B7—B8	−104.18 (10)
C10—C9—N3—O3B	−6.26 (19)	B1—B4—B8—B10	−81.44 (15)
C8—C9—N3—O3A	−6.58 (19)	B7—B4—B8—B10	22.89 (11)
C10—C9—N3—O3A	173.61 (13)	B3—B4—B8—B10	−21.59 (15)
C1—N1—B2—B1	−47.65 (19)	B5—B4—B8—B10	−104.86 (13)
C5—N1—B2—B1	125.96 (13)	B1—B4—B8—B9	−21.70 (15)
C1—N1—B2—B9	167.10 (13)	B7—B4—B8—B9	82.63 (10)
C5—N1—B2—B9	−19.29 (16)	B3—B4—B8—B9	38.14 (12)
C1—N1—B2—B6	97.94 (15)	B5—B4—B8—B9	−45.12 (9)
C5—N1—B2—B6	−88.46 (13)	B1—B4—B8—B5	23.42 (11)
C1—N1—B2—B3	24.4 (2)	B7—B4—B8—B5	127.75 (11)
C5—N1—B2—B3	−162.03 (12)	B3—B4—B8—B5	83.27 (11)
C1—N1—B2—B5	−119.62 (16)	B1—B4—B8—B7	−104.33 (13)
C5—N1—B2—B5	53.98 (18)	B3—B4—B8—B7	−44.49 (10)
B4—B1—B2—N1	−179.91 (11)	B5—B4—B8—B7	−127.75 (11)
B3—B1—B2—N1	122.24 (13)	B1—B5—B8—B10	80.67 (14)
B5—B1—B2—N1	−121.88 (13)	B2—B5—B8—B10	21.70 (14)
B4—B1—B2—B9	−33.61 (14)	B9—B5—B8—B10	−23.30 (10)
B3—B1—B2—B9	−91.46 (12)	B4—B5—B8—B10	103.64 (12)
B5—B1—B2—B9	24.42 (11)	B1—B5—B8—B9	103.97 (12)
B4—B1—B2—B6	33.70 (14)	B2—B5—B8—B9	45.01 (9)
B3—B1—B2—B6	−24.16 (11)	B4—B5—B8—B9	126.94 (11)
B5—B1—B2—B6	91.73 (12)	B1—B5—B8—B4	−22.97 (11)
B4—B1—B2—B3	57.86 (10)	B2—B5—B8—B4	−81.93 (10)
B5—B1—B2—B3	115.89 (11)	B9—B5—B8—B4	−126.94 (11)
B4—B1—B2—B5	−58.03 (11)	B1—B5—B8—B7	21.25 (14)
B3—B1—B2—B5	−115.89 (11)	B2—B5—B8—B7	−37.71 (12)
B4—B1—B3—B2	−112.89 (11)	B9—B5—B8—B7	−82.72 (10)
B5—B1—B3—B2	−55.44 (10)	B4—B5—B8—B7	44.23 (10)
B4—B1—B3—B7	−23.54 (11)	B3—B7—B8—B10	−109.60 (12)
B2—B1—B3—B7	89.35 (11)	B4—B7—B8—B10	−154.69 (12)
B5—B1—B3—B7	33.91 (14)	B6—B7—B8—B10	−50.17 (9)
B4—B1—B3—B6	−89.42 (12)	B10—B7—B8—B9	50.51 (9)
B2—B1—B3—B6	23.47 (11)	B3—B7—B8—B9	−59.09 (10)
B5—B1—B3—B6	−31.97 (15)	B4—B7—B8—B9	−104.18 (10)
B2—B1—B3—B4	112.89 (11)	B6—B7—B8—B9	0.34 (9)
B5—B1—B3—B4	57.45 (10)	B10—B7—B8—B4	154.69 (12)
N1—B2—B3—B1	−103.53 (16)	B3—B7—B8—B4	45.09 (10)
B9—B2—B3—B1	110.09 (12)	B6—B7—B8—B4	104.52 (10)
B6—B2—B3—B1	154.75 (12)	B10—B7—B8—B5	109.80 (12)
B5—B2—B3—B1	49.94 (10)	B3—B7—B8—B5	0.21 (12)
N1—B2—B3—B7	147.20 (13)	B4—B7—B8—B5	−44.89 (10)
B1—B2—B3—B7	−109.27 (12)	B6—B7—B8—B5	59.63 (10)
B9—B2—B3—B7	0.82 (12)	N1—B2—B9—B10	−131.89 (12)
B6—B2—B3—B7	45.48 (10)	B1—B2—B9—B10	80.93 (14)
B5—B2—B3—B7	−59.33 (11)	B6—B2—B9—B10	−24.71 (11)
N1—B2—B3—B6	101.72 (15)	B3—B2—B9—B10	20.03 (14)
B1—B2—B3—B6	−154.75 (12)	B5—B2—B9—B10	104.75 (13)
B9—B2—B3—B6	−44.66 (10)	N1—B2—B9—B8	168.73 (11)
B5—B2—B3—B6	−104.81 (10)	B1—B2—B9—B8	21.55 (14)
N1—B2—B3—B4	−153.98 (14)	B6—B2—B9—B8	−84.09 (10)
B1—B2—B3—B4	−50.45 (10)	B3—B2—B9—B8	−39.35 (12)
B9—B2—B3—B4	59.64 (11)	B5—B2—B9—B8	45.37 (10)
B6—B2—B3—B4	104.30 (10)	N1—B2—B9—B5	123.36 (13)
B5—B2—B3—B4	−0.51 (10)	B1—B2—B9—B5	−23.82 (11)
B2—B1—B4—B7	−33.46 (14)	B6—B2—B9—B5	−129.46 (11)
B3—B1—B4—B7	23.61 (11)	B3—B2—B9—B5	−84.72 (11)
B5—B1—B4—B7	−90.62 (12)	N1—B2—B9—B6	−107.18 (12)
B2—B1—B4—B8	33.20 (14)	B1—B2—B9—B6	105.64 (12)
B3—B1—B4—B8	90.27 (12)	B3—B2—B9—B6	44.74 (10)
B5—B1—B4—B8	−23.95 (11)	B5—B2—B9—B6	129.46 (11)
B2—B1—B4—B3	−57.07 (9)	B4—B8—B9—B10	−109.00 (11)
B5—B1—B4—B3	−114.23 (10)	B5—B8—B9—B10	−154.48 (11)
B2—B1—B4—B5	57.16 (10)	B7—B8—B9—B10	−50.25 (9)
B3—B1—B4—B5	114.23 (10)	B10—B8—B9—B2	109.17 (11)
B2—B3—B4—B1	50.92 (9)	B4—B8—B9—B2	0.17 (12)
B7—B3—B4—B1	154.65 (11)	B5—B8—B9—B2	−45.31 (9)
B6—B3—B4—B1	109.46 (11)	B7—B8—B9—B2	58.92 (10)
B1—B3—B4—B7	−154.65 (11)	B10—B8—B9—B5	154.48 (11)
B2—B3—B4—B7	−103.73 (10)	B4—B8—B9—B5	45.48 (10)
B6—B3—B4—B7	−45.19 (9)	B7—B8—B9—B5	104.23 (10)
B1—B3—B4—B8	−109.43 (12)	B10—B8—B9—B6	49.91 (9)
B2—B3—B4—B8	−58.51 (11)	B4—B8—B9—B6	−59.09 (11)
B7—B3—B4—B8	45.22 (10)	B5—B8—B9—B6	−104.57 (10)
B6—B3—B4—B8	0.04 (13)	B7—B8—B9—B6	−0.34 (9)
B1—B3—B4—B5	−50.42 (9)	B1—B5—B9—B10	−79.76 (14)
B2—B3—B4—B5	0.50 (9)	B2—B5—B9—B10	−103.02 (12)
B7—B3—B4—B5	104.24 (10)	B8—B5—B9—B10	23.29 (11)
B6—B3—B4—B5	59.05 (11)	B4—B5—B9—B10	−21.36 (14)
B4—B1—B5—B2	112.78 (11)	B1—B5—B9—B2	23.26 (11)
B3—B1—B5—B2	55.43 (10)	B8—B5—B9—B2	126.31 (10)
B4—B1—B5—B8	23.76 (11)	B4—B5—B9—B2	81.66 (10)
B2—B1—B5—B8	−89.02 (12)	B1—B5—B9—B8	−103.05 (13)
B3—B1—B5—B8	−33.59 (14)	B2—B5—B9—B8	−126.31 (10)
B4—B1—B5—B9	88.92 (12)	B4—B5—B9—B8	−44.66 (10)
B2—B1—B5—B9	−23.85 (11)	B1—B5—B9—B6	−19.93 (15)
B3—B1—B5—B9	31.57 (14)	B2—B5—B9—B6	−43.19 (10)
B2—B1—B5—B4	−112.78 (11)	B8—B5—B9—B6	83.12 (10)
B3—B1—B5—B4	−57.35 (10)	B4—B5—B9—B6	38.47 (12)
N1—B2—B5—B1	103.70 (15)	B2—B6—B9—B10	152.64 (12)
B9—B2—B5—B1	−154.29 (12)	B3—B6—B9—B10	108.96 (12)
B6—B2—B5—B1	−110.36 (11)	B7—B6—B9—B10	50.37 (9)
B3—B2—B5—B1	−49.90 (9)	B10—B6—B9—B2	−152.64 (12)
N1—B2—B5—B8	−147.21 (13)	B3—B6—B9—B2	−43.68 (10)
B1—B2—B5—B8	109.10 (12)	B7—B6—B9—B2	−102.27 (10)
B9—B2—B5—B8	−45.19 (9)	B10—B6—B9—B8	−50.02 (9)
B6—B2—B5—B8	−1.26 (12)	B2—B6—B9—B8	102.62 (10)
B3—B2—B5—B8	59.19 (10)	B3—B6—B9—B8	58.94 (11)
N1—B2—B5—B9	−102.01 (15)	B7—B6—B9—B8	0.34 (9)
B1—B2—B5—B9	154.29 (12)	B10—B6—B9—B5	−109.36 (12)
B6—B2—B5—B9	43.93 (9)	B2—B6—B9—B5	43.28 (10)
B3—B2—B5—B9	104.38 (10)	B3—B6—B9—B5	−0.40 (13)
N1—B2—B5—B4	154.11 (13)	B7—B6—B9—B5	−59.00 (11)
B1—B2—B5—B4	50.41 (9)	B4—B8—B10—B9	90.44 (12)
B9—B2—B5—B4	−103.88 (10)	B5—B8—B10—B9	23.96 (11)
B6—B2—B5—B4	−59.94 (10)	B7—B8—B10—B9	113.94 (10)
B3—B2—B5—B4	0.51 (9)	B9—B8—B10—B6	−56.60 (9)
B7—B4—B5—B1	109.29 (12)	B4—B8—B10—B6	33.84 (14)
B8—B4—B5—B1	154.30 (12)	B5—B8—B10—B6	−32.64 (13)
B3—B4—B5—B1	50.43 (9)	B7—B8—B10—B6	57.33 (10)
B1—B4—B5—B2	−50.93 (9)	B9—B8—B10—B7	−113.94 (10)
B7—B4—B5—B2	58.36 (11)	B4—B8—B10—B7	−23.49 (11)
B8—B4—B5—B2	103.37 (10)	B5—B8—B10—B7	−89.98 (12)
B3—B4—B5—B2	−0.50 (9)	B2—B9—B10—B8	−89.45 (11)
B1—B4—B5—B8	−154.30 (12)	B5—B9—B10—B8	−23.94 (11)
B7—B4—B5—B8	−45.02 (10)	B6—B9—B10—B8	−114.82 (10)
B3—B4—B5—B8	−103.87 (10)	B2—B9—B10—B6	25.38 (11)
B1—B4—B5—B9	−109.50 (12)	B8—B9—B10—B6	114.82 (10)
B7—B4—B5—B9	−0.21 (13)	B5—B9—B10—B6	90.88 (11)
B8—B4—B5—B9	44.81 (10)	B2—B9—B10—B7	−31.89 (14)
B3—B4—B5—B9	−59.07 (11)	B8—B9—B10—B7	57.55 (10)
N1—B2—B6—B10	132.36 (12)	B5—B9—B10—B7	33.62 (14)
B1—B2—B6—B10	−80.28 (14)	B6—B9—B10—B7	−57.27 (10)
B9—B2—B6—B10	24.67 (10)	B2—B6—B10—B8	31.12 (13)
B3—B2—B6—B10	−103.74 (12)	B9—B6—B10—B8	56.38 (9)
B5—B2—B6—B10	−19.16 (13)	B3—B6—B10—B8	−33.73 (13)
N1—B2—B6—B9	107.69 (12)	B7—B6—B10—B8	−57.59 (10)
B1—B2—B6—B9	−104.95 (13)	B2—B6—B10—B9	−25.26 (10)
B3—B2—B6—B9	−128.41 (11)	B3—B6—B10—B9	−90.11 (11)
B5—B2—B6—B9	−43.83 (9)	B7—B6—B10—B9	−113.97 (11)
N1—B2—B6—B3	−123.90 (13)	B2—B6—B10—B7	88.71 (11)
B1—B2—B6—B3	23.46 (11)	B9—B6—B10—B7	113.97 (11)
B9—B2—B6—B3	128.41 (11)	B3—B6—B10—B7	23.86 (11)
B5—B2—B6—B3	84.58 (11)	B3—B7—B10—B8	89.79 (12)
N1—B2—B6—B7	−168.65 (10)	B4—B7—B10—B8	23.47 (11)
B1—B2—B6—B7	−21.29 (13)	B6—B7—B10—B8	114.01 (11)
B9—B2—B6—B7	83.66 (10)	B3—B7—B10—B9	32.88 (14)
B3—B2—B6—B7	−44.75 (9)	B4—B7—B10—B9	−33.43 (14)
B5—B2—B6—B7	39.83 (11)	B6—B7—B10—B9	57.10 (10)
B1—B3—B6—B10	80.18 (14)	B8—B7—B10—B9	−56.90 (10)
B2—B3—B6—B10	103.08 (12)	B3—B7—B10—B6	−24.22 (11)
B7—B3—B6—B10	−23.35 (10)	B4—B7—B10—B6	−90.53 (12)
B4—B3—B6—B10	21.49 (14)	B8—B7—B10—B6	−114.01 (11)
B1—B3—B6—B2	−22.90 (11)		
